# Impact-Driven Energy Harvesting: Piezoelectric Versus Triboelectric Energy Harvesters

**DOI:** 10.3390/s20205828

**Published:** 2020-10-15

**Authors:** Panu Thainiramit, Phonexai Yingyong, Don Isarakorn

**Affiliations:** Department of Instrumentation and Control Engineering, Faculty of Engineering, King Mongkut’s Institute of Technology Ladkrabang, Bangkok 10520, Thailand; panu.th@kmitl.ac.th (P.T.); 62601019@kmitl.ac.th (P.Y.)

**Keywords:** piezoelectric energy harvesting, triboelectric energy harvesting, low-frequency vibration energy harvesting, direct-force generator

## Abstract

This work investigated the mechanical and electrical behaviors of piezoelectric and triboelectric energy harvesters (PEHs and TEHs, respectively) as potential devices for harvesting impact-driven energy. PEH and TEH test benches were designed and developed, aiming at harvesting low-frequency mechanical vibration generated by human activities, for example, a floor-tile energy harvester actuated by human footsteps. The electrical performance and behavior of these energy harvesters were evaluated and compared in terms of absolute energy and power densities that they provided and in terms of these energy and power densities normalized to unit material cost. Several aspects related to the design and development of PEHs and TEHs as the energy harvesting devices were investigated, covering the following topics: construction and mechanism of the energy harvesters; electrical characteristics of the fabricated piezoelectric and triboelectric materials; and characterization of the energy harvesters. At a 4 mm gap width between the cover plate and the stopper (the mechanical actuation components of both energy harvesters) and a cover plate pressing frequency of 2 Hz, PEH generated 27.64 mW, 1.90 mA, and 14.39 V across an optimal resistive load of 7.50 kΩ, while TEH generated 1.52 mW, 8.54 µA, and 177.91 V across an optimal resistive load of 21 MΩ. The power and energy densities of PEH (4.57 mW/cm^3^ and 475.13 µJ/cm^3^) were higher than those of TEH (0.50 mW/cm^3^, and 21.55 µJ/cm^3^). However, when the material cost is taken into account, TEH provided higher power and energy densities per unit cost. Hence, it has good potential for upscaling, and is considered well worth the investment. The advantages and disadvantages of PEH and TEH are also highlighted as main design factors.

## 1. Introduction

Investigation of energy harvesting from the ambient environment is increasing rapidly as a result of today’s efficient energy consumption requirement and a need for low environmental impact. Energy sources to be harvested are usually waste energy from activities such as machine vibration [1], transportation [2], human motion [3], wind flow [4], and ocean wave [5]. This source of direct force acting on the generators is the most promising candidate for harvesting energy for self-powered electronic and sensing systems [6]. As they are waste energy, they do not affect the environment any more negatively than when they are left as waste and are not getting harvested. Harvesting waste energy does not disrupt the local ecosystem and does not cause any pollution or global warming [7]. For this work, the kinetic energy source was mechanical vibration from human activities. Although the electrical output of waste energy harvesting is less than other alternative energy sources, it is sufficient for powering small electronic devices, which are rapidly being introduced for many applications in people’s daily routine [8].

There are many energy harvesting techniques, such as piezoelectric, triboelectric, pyroelectric, and electromagnetic transductions [8,9]. Piezoelectric energy harvesters (PEHs) and triboelectric energy harvesters (TEHs) have gained much attention in the last decade, as demonstrated by a large number of studies on the conversion of mechanical vibration energy to electrical energy [9,10]. Several of those were about the characterization and optimization of piezoelectric materials for energy harvesting, sensing, and actuating. For example, there has been a study on piezoelectric material on various substrates for a specific purpose, such as achieving small size, a higher degree of flexibility, being more stretchable, possessing a desired degree of roughness, and low cost [11]. Phosy Panthongsy et al. [12] proposed two different piezoelectric test benches. The piezoelectric material was used in the form of a cantilever, of which one end was attached to a holder on the base, and the other end was attached to a freely moving proof mass. The first test bench generated its electrical output by having the proof mass magnetize an iron bar on the cover plate to produce stress on the cantilever. The cantilever then freely oscillated after the cover plate was pressed and released. That test bench had a complex structure and was designed to prevent the cantilever from over-bending. On the other hand, the second type of test bench had a simple structure and a higher electrical performance than the first type because electrical energy was generated each time the cover plate was pressed and each time it was released. In another study, Don Isarakorn et al. [13] investigated the behavior of double-stage energy harvesting floor tile. They varied the excitation acceleration and gap width of the cover plate, observed the results, and found that the accelerations of the moving cover plate and gap width were directly proportional to the electrical output. Although that harvester was not designed for harvesting energy at their resonance frequency, it had a potential to harvest energy from low and variable frequency mechanical vibration sources, for instance, human motion [14].

A triboelectric energy harvester converts mechanical energy to electrical energy from friction or temporary contact between two different triboelectric materials. Its basic energy harvesting mechanism is based on combined contact electrification and electrostatic induction [15]. TEH is a promising technology that has attracted much attention and developed rapidly [16,17,18,19,20]. Four basic energy harvesting modes of TEHs are vertical contact-separation (CS) mode, lateral-sliding (LS) mode, single-electrode (SE) mode, and freestanding triboelectric-layer (FT) mode [21]. Examples of studies on characterization and optimization of the electrical performance of triboelectric and electrode materials, device fabrication, and environment condition control are the following studies [22,23,24,25,26,27]. In addition, Cun Xin Lu et al. [28] investigated the temperature effect on the triboelectric electrical output of a single-electrode mode and reported that, at a low temperature, the TEH was able to generate high electrical output, and vice versa. The relative humidity is also a critical factor that affects triboelectric charge density. Therefore, a device properly installed in a controlled environment could be highly efficient [29]. Moreover, enlarging the size of a TEH and parallelly connecting many of them in the same device has the potential to increase the electrical output [30]. In addition, hybridizing TEH with another energy harvesting device that operates by a different harvesting mechanism has been investigated and found to increase energy production efficiency [31,32,33].

Even though there have been numerous research studies on the electrical output performance of PEHs and TEHs, including applications with an individual generator and combined generators, none of them have compared the voltages, powers, and energies provided by them as well as their produced energy densities and costs. Therefore, in this study, we evaluated and compared the electrical performance and behavior of TEH and PEH in terms of their electrical output density per material cost. This development of energy harvester design based on low-frequency vibration for harvesting mechanical energy from human motion or activities had the potential to be used in practical applications and is well worth the investment.

The rest of the paper is structured as follows. Section 2 describes the construction and working principles of both PEH and TEH test benches for harvesting energy from low-frequency mechanical vibration sources. Section 3 describes the details of the fabrication material of both PEHs and TEHs, as well as the experimental methods and setups. Section 4 presents the experimental results of both PEHs and TEHs, including the electrical output characteristics, the electrical performance comparison, and the potential of practical applications. Section 5 discusses the experimental results related to gap width, electrical performance, power density, energy density, unit cost consideration, and summary of the advantages and disadvantages. Lastly, Section 6 concludes the paper.

## 2. Construction and Mechanism of the Test Bench

The structures of the test benches—two different energy harvesters (PEH and TEH)—were designed and developed. Their mechanisms, the measurement schematic, and their output voltage characteristics are described in Section 2.1 and Section 2.2, respectively.

### 2.1. Piezoelectric Energy Harvester Test Bench

**Figure 1:**
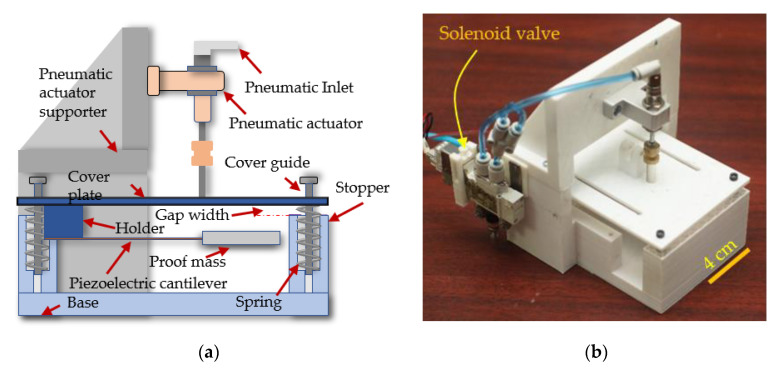
Piezoelectric energy harvester (PEH) test bench: (**a**) cross-section of PEH; (**b**) photograph of PEH test bench.

**Figure 2:**
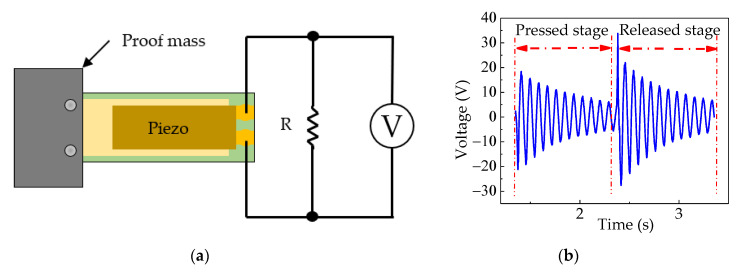
(**a**) Measurement setup of PEH, and (**b**) typical output voltage of PEH test bench.

The purpose of the piezoelectric energy harvester test bench was to harvest waste energy from mechanical vibration at a low frequency into usable electrical energy. The cover plate of the test bench absorbed the excitation impact from a pneumatic actuator. A pressure valve regulator was used to control the compressed air pressure into the pneumatic actuator. Springs were used to separate the cover plate from the stopper of the base and to store the mechanical energy after the cover plate was pressed, then the mass tip of the cantilever bounced and oscillated freely in the air, as shown in Figure 1a, and the images of actual PEH test bench are shown in Figure 1b. The essential element for energy conversion was the piezoelectric cantilever. One end of it was side-mounted to a holder on the cover plate, and the other end was attached to a proof mass. This cantilever generates electricity in two stages. The first stage was when the cover plate was pressed. The strain within the beam caused by the pressing of the cover plate allows the cantilever beam to oscillate freely afterward. After the cover plate was pressed by an actuator, the stress in the cover plate spring pushes it back upward. The mass tip of the piezoelectric cantilever sprung upward, causing the beam to bend and freely oscillate until the next actuating period. This occurrence caused another round of electricity generation, as shown in Figure 2.

### 2.2. Triboelectric Energy Harvester Test Bench

**Figure 3:**
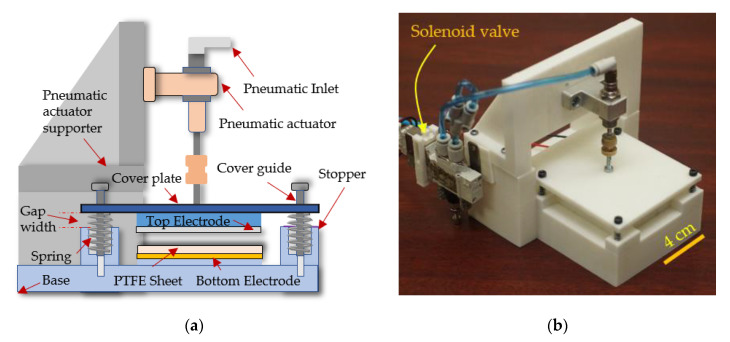
Triboelectric energy harvester (TEH) test bench: (**a**) Cross-section of TEH test bench; (**b**) Photograph of TEH test bench. PTFE, polytetrafluoroethylene.

The primary electricity generation mechanism of the triboelectric energy harvester was charge accumulation by friction or by temporary contact between two different triboelectric materials. The design and operation of the TEH test bench design were not very different from those of the PEH test bench. The top electrode mounted under the cover plate absorbed direct force from the pneumatic actuator. The pneumatic pressure and the pressing frequency applied to the pneumatic actuator were controlled by a pressure valve regulator and a function generator, respectively. The base supported the cover guide, the springs, and the pneumatic actuator supporter, as shown in Figure 3a, and the images of the actual TEH test bench is shown in Figure 3b. In this research, the TEH test bench was a contact mode single-electrode triboelectric energy harvester. The electrical output is generated by pressing the cover plate downward; subsequently, the top electrode contacted the triboelectric material, and the contact electrification created tribo-charges on the surface of the triboelectric material attached to the bottom electrode and on the top electrode, which was also a triboelectric material; then, the charges were transferred from the bottom to the top electrode through an external circuit as a result of electrostatic transduction [15].

**Figure 4:**
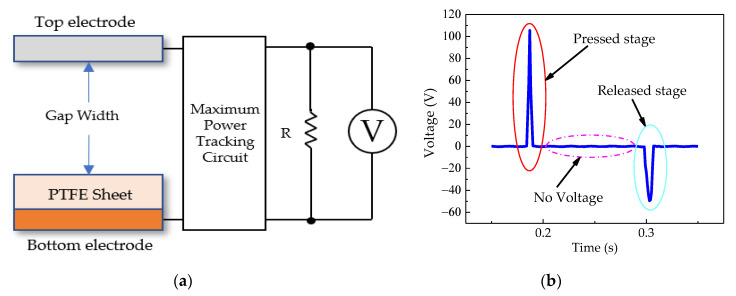
(**a**) Measurement setup of TEH, and (**b**) typical output voltage of TEH.

Figure 4a shows the measurement setup, including the TEH source connected to the maximum power tracking (MTP) circuit, for measuring the electrical characteristics across the resistive loads [34]. The TEH had a very high output impedance, so the output resistive load had a very high resistance as well. When the resistive load was higher than a measurement probe of the oscilloscope, the TEH output power could not be determined accurately. This MPT technique was thus applied to measure the output power. The output voltage generation occurred in two stages: a pressed-stage and a released-stage, as shown in Figure 4b.

## 3. Materials and Methods

The types, sizes, costs, and critical properties of the fabricated PEH and TEH materials used in this work are reported below as well as the experimental methods and setup.

### 3.1. Fabrication Materials

A piezoelectric material, we used a Midé S230-J1FR-1808XB piezoelectric module (7.1 × 2.54 × 0.076 cm^3^) in the piezoelectric cantilever configuration. One end of it was attached to a holder at the cover plate, and the other end was mounted with a proof mass (stainless-steel, 3 × 2.6 × 0.6 cm^3^, total mass 40 g), and total PEH volume was 6.05 cm^3^. The cost of this bimorph cantilever was US$274 per unit, discounted if bought in a large quantity. For example, for 100 units, the price per unit was only US$82 [35]. The physical and mechanical properties of this piezoelectric cantilever are shown in Table 1 [36]. 

For the TEH test bench, Wang et al. [37] listed the materials in the triboelectric series. A material will reach more negative charge when touching a material to the bottom of the series. The farther away two materials were selected from each other on the series, the greater charge will be transferred. Aluminium appears near the top of the series, whereas polytetrafluoroethylene (PTFE) is near the bottom, so Al and PTFE were chosen for this project. A PTFE sheet (5 × 5 × 0.1 cm^3^) is attached to the bottom electrode (primary electrode), and the assembly is mounted on the base of the test bench. The bottom electrode (5 × 5 × 0.01 cm^3^) is a copper foil (MT 8113C copper foil tape conductive adhesive). The top electrode (5 × 5 × 0.012 cm^3^) is an aluminum foil (3M 425 Aluminum foil tape) mounted on a supporter on the cover plate, and the total volume of TEH was 3.05 cm^3^. This top electrode functioned both as a triboelectric material and a reference electrode. The average price of a PTFE sheet, including the top electrode and bottom electrode, was US$1.44. In a large quantity, the price could be discounted to US$1.17 per square centimeter. The physical and mechanical properties of the PTFE are shown in Table 2 [38].

### 3.2. Experimental Set-up and Methods

This section aims to present an overview of the experimental setup, including the mechanical energy input, which was a critical part of our direct mechanical force applied to an energy harvesting device.

The mechanical energy input that we used was a constant air pressure of 600 kPa from a pneumatic pump, regulated by a pressure valve regulator. The compressed air pressure was the pneumatic actuator (CJPB6-15 from SMC Corporation) that provided a direct mechanical force 17.20 N on the cover plate of both types of test benches, and a spring, with the spring constant of 263 N/m, was used for separating the cover plate from the stopper. The acceleration of the moving cover plate was measured with an accelerometer (EI-CALC). The frequency of the application of direct force was controlled by a function generator (GW INSTEK AFG-2225), which could be set to be any desired frequency that was in harmony with the low-frequency actuation of the mechanical parts of both types of test benches. An oscilloscope (Tektronix TDS-3032B) was used to measure the open-circuit voltage and the output voltage across resistive loads through an MPT circuit, as shown in Figure 4a. Both PEHs and TEHs have experimented at room temperature.

The equations related to this experiment are explained below. For calculating the output power (P) for optimal load identification, the equation was Equation (1). In Equation (1), $V_{L}$ was the output voltage across the resistive loads $R_{L}$. For calculating the total energy harvested $E(t_{n})$, the equation was Equation (2), where $t_{n}$ was the total time while the triboelectric was working $V_{L(t_{M})}$ was the voltage across the resistive load at a time $t_{m}$ and Δt was the sampling time interval. We used these equations in the same way that they were used in [12,13]:

(1) $$P=\frac{{V_{L}}^{2}}{R_{L}}$$

and

(2) $$E\left(t_{n}\right)=\sum_{m=1}^{n}\frac{{V_{L}}^{2}\left(t_{m}\right)}{R_{L}}\Delta t,\;\mathrm{for}\;n>0$$

We divided the power and energy obtained from Equations (1) and (2) with the volume and cost of material of each of the two energy harvesters to determine and compare their power and energy densities as well as cost-effectiveness.

The procedural steps in the experiments of both PEH and TEH are as follows.

(1) Designing and fabricating test benches that used the same pneumatic actuator to apply a proper direct force to their cover plate and doing the same thing for the spring that pushed the cover plate away from the stopper. Images of actual PEH and TEH test benches are shown in Figure 1b and Figure 3b, respectively.
(2) Using resistive loads to connect the top electrode to the bottom electrode to identify their optimal load and peak power by measuring the peak output voltage across resistive load while varying the gap width between the cover plate and the stopper from 1 mm to 4 mm.
(3) Measuring and recording the output voltages of PEH and TEH for a 4 mm gap width while varying the cover plate pressing frequency from 0.5 Hz to 5 Hz in an increment of 0.5. The measured voltages were used in energy computation.
(4) Calculating the electrical performances of the PEH and TEH normalized by material volume and cost into output voltage, power density, and energy density per unit cost, which would be immensely useful for designing and developing a practical energy harvesting device.

## 4. Experimental Results

The PEH and TEH converted mechanical energy into electrical energy by deformation of its cantilever and by making temporary contact between triboelectric materials, respectively. It was difficult to compare most of the conventional parameters related to energy harvester, so we focused on their practical utility; we compared their power and energy densities. We applied an optimal input of direct force to the cover plate of each type of test bench and measured the electrical output. For proper comparison, we used the same input for exciting the cover plates of both types of the test bench, as well as the same pneumatic pressure and gap width between the cover plate and the stopper; the initial acceleration of the moving cover plate was 0.93 *g*. At gap widths of 1, 2, 3, and 4 mm, the acceleration when the cover plate impacted on the stopper was 3.05 *g*, 3.77 *g*, 4.06 *g*, and 4.28 *g*, respectively. The *g* is the gravitational acceleration, where 1 *g* implies 9.81 m/s^2^. The electrical performances of the PEH and TEH are presented in Section 4.1 and Section 4.2, respectively. The experimental results of the two techniques in various scenarios are compared and discussed in Section 4.3.

### 4.1. Electrical Output Characteristics of PEH Test Bench

**Figure 5:**
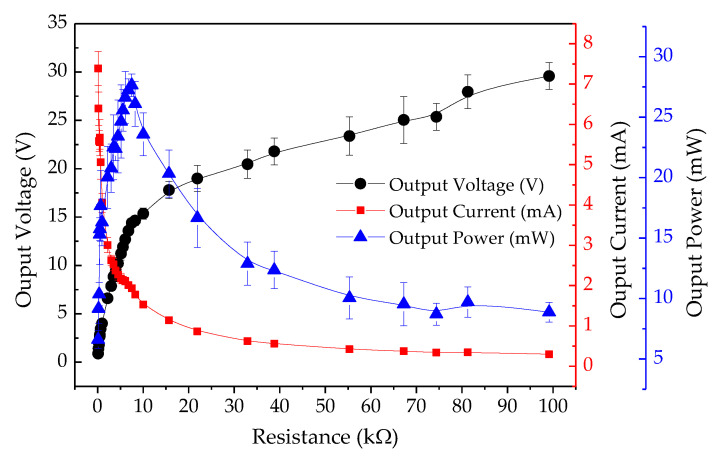
Electrical output characteristics of PEH across resistive loads, at 4 mm gap width.

**Figure 6:**
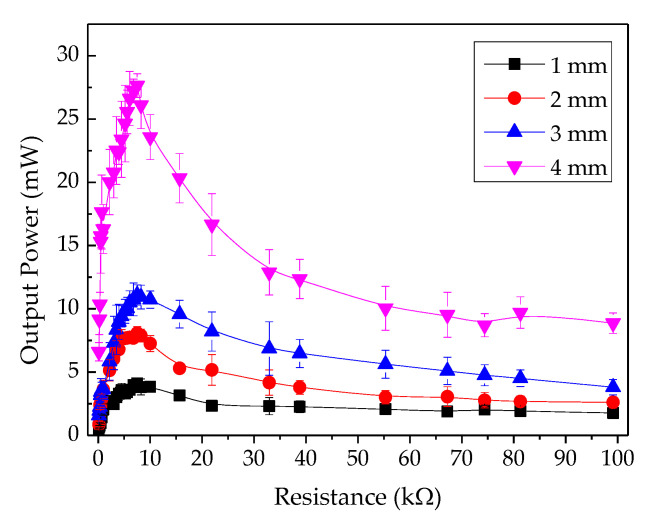
Output power at various gap widths from 1 mm to 5 mm.

The electrical performance of the PEH test bench was determined by connecting resistive loads between the top electrode and the bottom electrode mounted on the piezoelectric cantilever. Figure 5 displays the electrical output characteristics across variable resistive loads. A constant pneumatic pressure was applied to the cover plate under the condition of a 4 mm gap width between the cover plate and the stopper. The output current was directly proportional to the output voltage across the resistive loads. The peak output voltage, peak output current, and peak output power across 7.50 kΩ optimal loads were 14.39 V, 1.92 mA, and 27.64 mW, respectively. Figure 6 shows the output power under the conditions of 1–4 mm gap width. The peak output powers across the optimal load were found to be 4.12 mW, 8.07 mW, 11.10 mW, and 27.64 mW, respectively. Therefore, it was clear that the output power was directly proportional to the gap width between the cover plate and the stopper.

**Figure 7:**
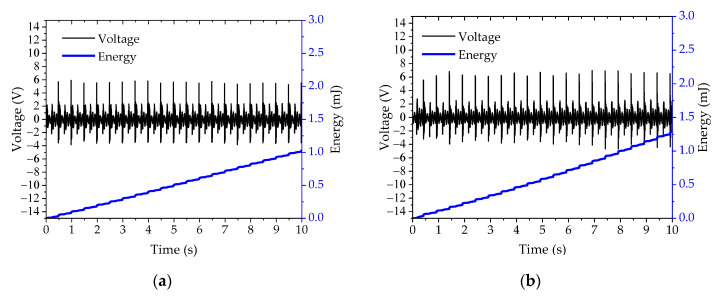

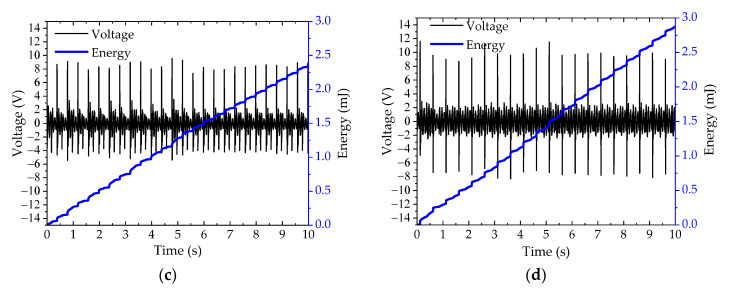
Output voltage and energy curve of PEH across the optimal load at 2 Hz with varying gap widths of (**a**) 1 mm, (**b**) 2 mm, (**c**) 3 mm, and (**d**) 4 mm.

Figure 7 shows the output voltage and energy across the optimal load at 10 s after the cover plate was actuated and at four different gap widths from 1 mm to 4 mm under the pressing frequency of 2 Hz. The output voltage and energy tended to slightly increase when the gap width was varied from 1 mm to 4 mm. At gap widths of 1, 2, 3, and 4 mm, the average peak voltage and total energy are 6 V and 1.03 mJ, 7 V and 1.29 mJ, 8 V and 2.37 mJ, and 9 V and 2.88 mJ, respectively.

### 4.2. Electrical Output Characteristics of TEH Test Bench

**Figure 8:**
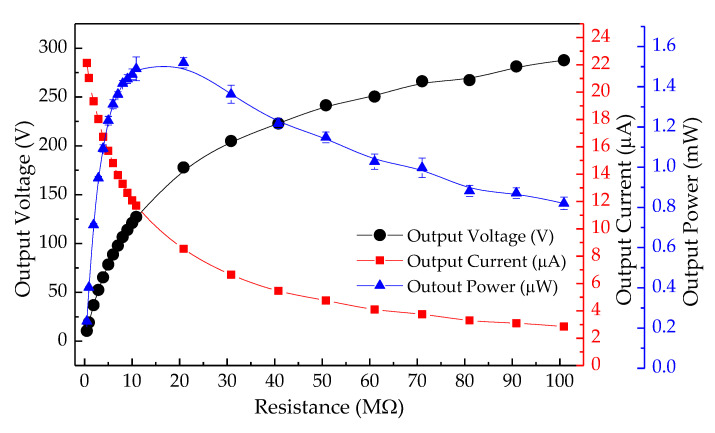
Electrical output parameters of TEHs across resistive loads at 4 mm gap width.

**Figure 9:**
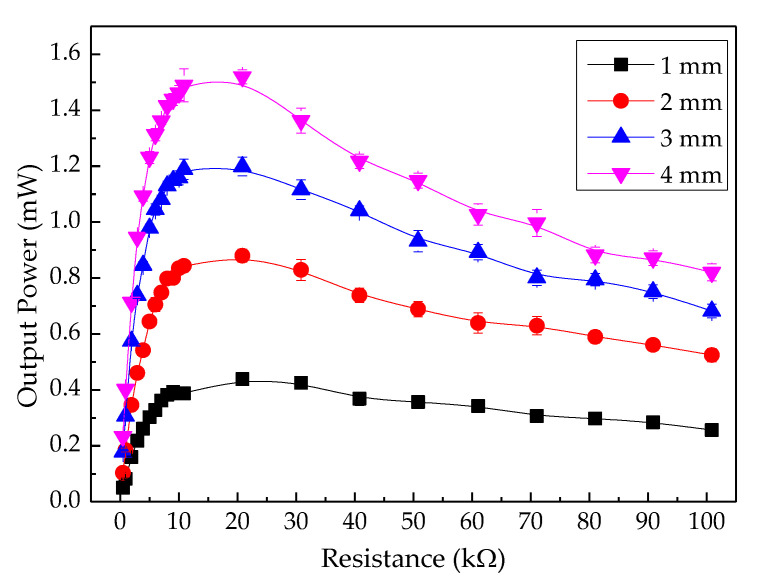
Output power of TEH test bench at various gap widths from 1 mm to 4 mm.

**Figure 10:**
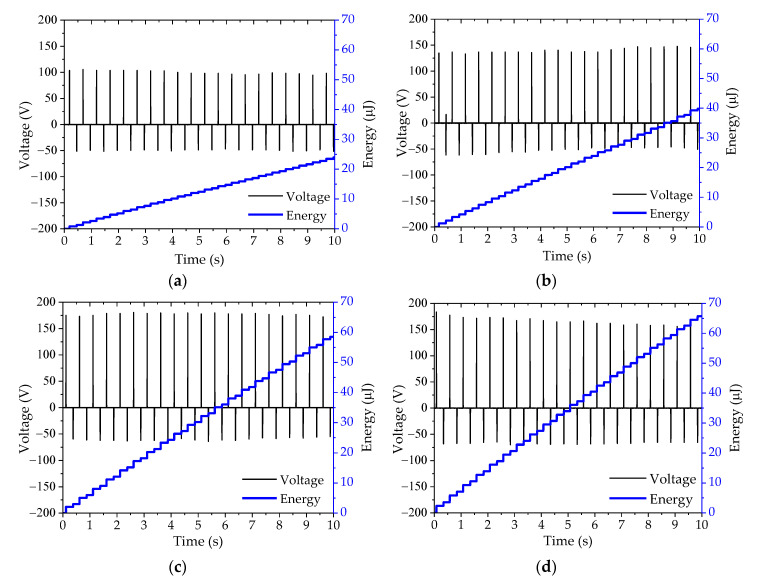
Output voltage and energy curve of TEH across the optimal load under 2 Hz with varying gap width; (**a**–**d**) are of 1 mm to 4 mm gap width in an increment of 1 mm.

The TEH test bench was designed to harvest energy from low frequency impacts, so the electrical performance of this device was investigated by applying a constant pneumatic pressure to produce a direct force on the cover plate. The electrical output parameters at 4 mm gap width are shown in Figure 8. The output currents were directly proportional to the output voltages across resistive loads, the same as those observed in PEH. TEH had an optimal load of 21 MΩ. Its peak output voltage, peak output current, and peak output power were 177.91 V, 8.54 µA, and 1.52 mW, respectively. Its electrical performance varied with the gap width. The peak output power varied with the velocity of the top electrode moving down to press on the contact on the PTFE sheet and the gap width. It slightly increased with gap width: 0.44 mW for 1 mm gap width, 0.88 mW for 2 mm, 1.20 mW for 3 mm, and 1.52 mW for 4 mm gap width, as shown in Figure 9. The output voltage and energy at four different gap widths at 2 Hz and at 10 s after actuation are shown in Figure 10. The average peak voltage and energy tended to increase slightly with gap width: 100 V and 23.96 µJ, 130 V and 39.84 µJ, 170 V and 58.51 µJ, and 175 V and 65.73 µJ for 1, 2, 3, and 4 mm gap width, respectively.

### 4.3. Electrical Output Characteristics of PEH and TEH Test Benches

PEHs and TEHs were readily accessible technology for energy harvesting. It was still not clear which one was better in terms of energy harvesting based on direct force excitation. The electrical output parameters determined were output voltage, power density, and energy density.

#### 4.3.1. Output Voltage Comparison

**Figure 11:**
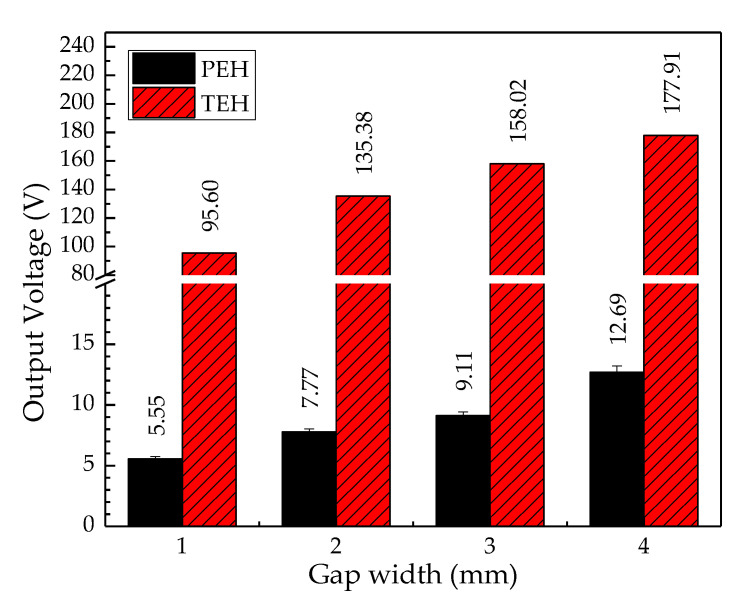
Output voltage of PEH and TEH.

Output voltage is a critical parameter for comparing PEH and TEH. The column graph in Figure 11 illustrates the peak output voltage across the optimal resistive load at different gap widths. It can be seen that the output voltage of both types of test bench was directly proportional to the gap width, and the output voltage at the gap widths from 1 mm to 3 mm of TEH were more than 17 times higher than the voltage density of PEH. At 4 mm gap width, the voltage density of TEH was 14 times higher than that of PEH. As an application may require a high operational voltage, TEH would be better than PEH for this kind of application.

#### 4.3.2. Power Density Comparison between PEH and TEH

**Figure 12:**
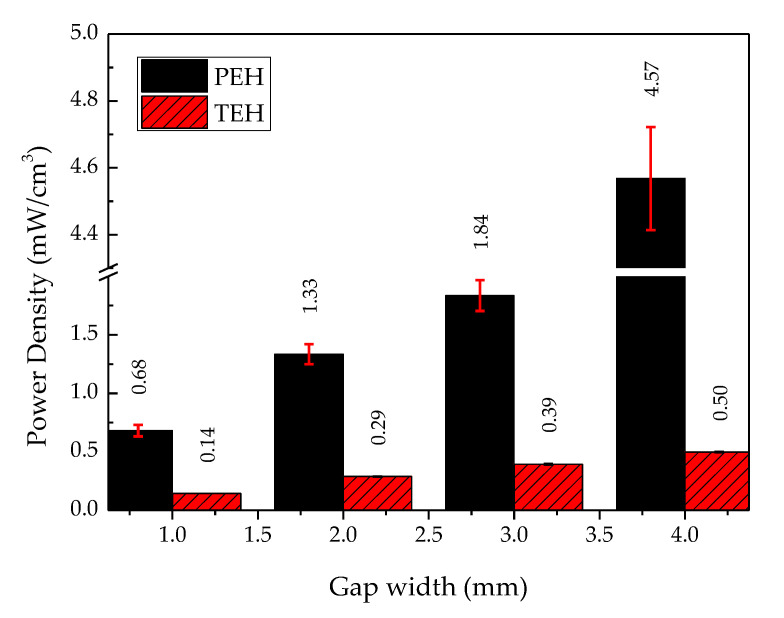
Power densities of PEH and TEH.

Figure 12 shows a column bar graph of the power density of PEH and TEH. The power density of PEH, for any gap widths, was higher than that of TEH. The power density of both devices was higher when the gap width was larger (between 1 and 4 mm). The power density of PEH at a gap width from 1 mm to 3 mm was more than four times higher than the power density of TEH at any gap width. At a 4 mm gap width, the power density of PEH was 9 times higher than that of TEH: 4.57 mW/cm^3^ for PEH and 0.50 mW/cm^3^ for TEH. PEH and TEH output powers are shown in Figure 6 and Figure 9. Both of them exhibited good potential for powering a small electronic device, of which some examples are shown in Table 3.

#### 4.3.3. Energy Density Comparison between PEH and TEH

The energy densities of both types of energy harvesting devices were investigated under the conditions of direct force application on the cover plate, of variable pressing frequency from 0.5 Hz to 5 Hz, and of an optimal load for PEH and TEH, which was 7.5 kΩ and 21 MΩ, respectively.

**Figure 13:**
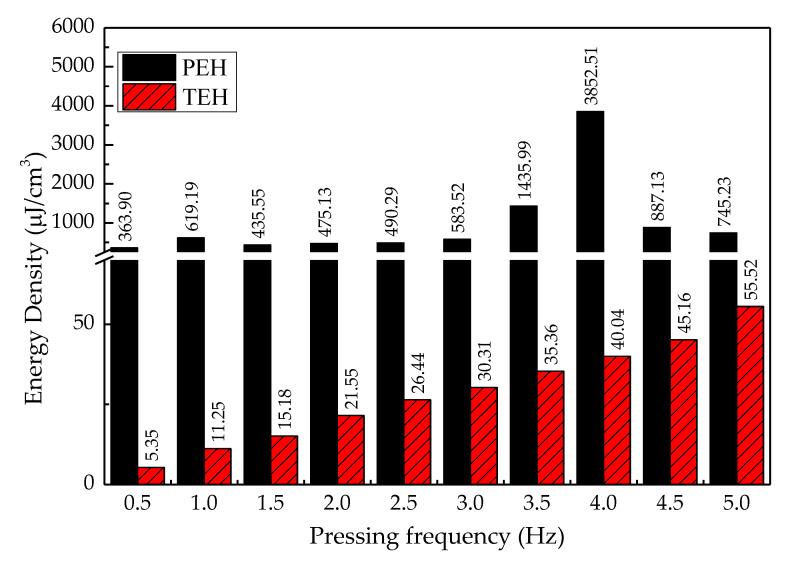
Energy densities of PEH and TEH under the variable pressing frequency of 0.5–5 Hz and at a 4 mm gap width.

Figure 13 shows the energy densities provided by PEH and TEH at a 4 mm gap width and under a variable pressing frequency between 0.5 and 5 Hz. It can be observed that PEH provided a higher energy density than TEH, under every pressing frequency from 0.5 to 5 Hz, in increments of 0.5 Hz.

The energy density of PEH slightly increased with input excitation frequency from 0.5 Hz to 3 Hz and reached the highest output energy density of 3.85 mJ/cm^3^ at the input pressing frequency of 4 Hz, but dropped dramatically at the excitation frequencies of 4.5 Hz and 5 Hz.

The resonance frequency of the cantilever in the PEH test bench was about 12 Hz, but we intended to harvest electrical energy from a source, providing a frequency of mechanical vibration that was lower than this resonance frequency—from the frequency of human steps, walking leisurely, at around 1–2 Hz. The varying energy density of PEH with pressing frequency might be the result of the harmonics of the direct-force excitation frequency. The outcome of this phenomenon can be observed in the dramatic decrease of peak output voltage as the moving cover plate stopped when it hit the stopper or the cover guide, as shown in Figure 7. The energy density of TEH increased linearly and reached the highest value of 55.52 µJ/cm^3^ at the highest pressing frequency of 5 Hz.

#### 4.3.4. Power Density and Energy Density per Unit Cost

**Figure 14:**
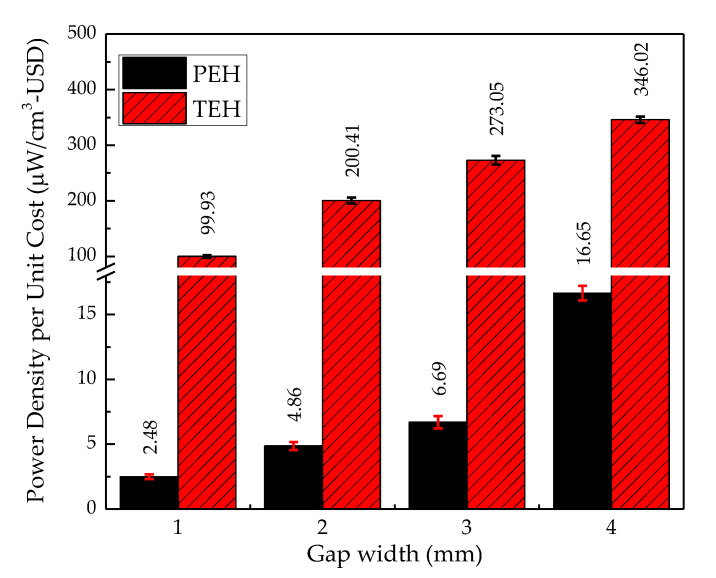
Power density per unit cost of PEH and TEH at a 4 mm gap width.

The cost of fabrication material is a critical parameter for the practical application of a device. In terms of price, TEH was much better than PEH, shown in Figure 14, but in terms of power and energy densities, PEH was more able than TEH. The power density per unit cost of TEH at a gap width from 1 mm to 3 mm was about 40 times higher than that of PEH, and the power density per unit cost of TEH at a 4 mm gap width was 20 times higher than that of PEH.

**Figure 15:**
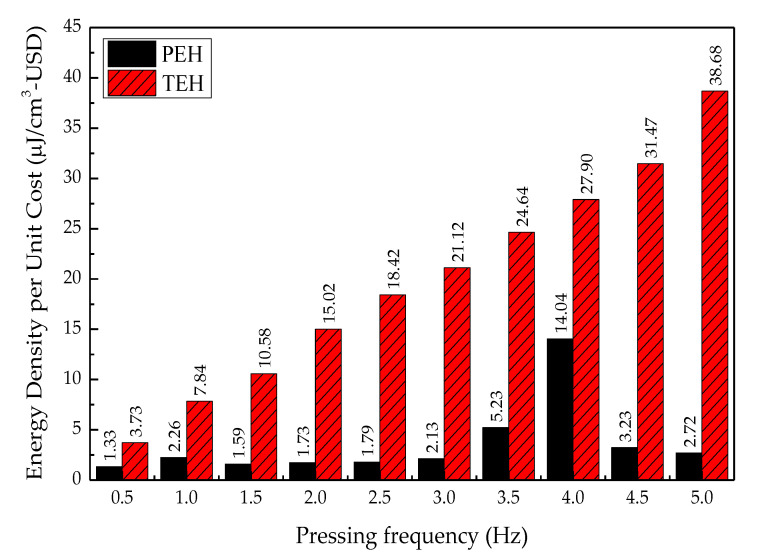
Energy density per unit cost of PEH and TEH at a 4 mm gap width.

Overall, the energy density per unit cost of TEH was much higher than that of PEH because triboelectric material for fabrication of TEH was much less expensive than piezoelectric material for fabrication of PEH. The energy density per unit cost of TEH increased linearly with the pressing frequency—38.68 µJ/cm^3^-USD at a 5 Hz pressing frequency. It was over 14 times higher than the energy density per unit cost of PEH. The energy density per unit cost of PEH reached the highest value of 14.04 µJ/cm^3^-USD at the 4 Hz pressing frequency, but it is still much lower than that of TEH, as shown in Figure 15.

## 5. Discussion

The electrical performances of a developed piezoelectric energy harvester (PEH) and a triboelectric energy harvester (TEH) actuated by direct mechanical force at a low frequency were investigated. In this section, we discuss three topics regarding the electrical performance outcomes: the relationship between the gap width separating the cover plate and the stopper and the electrical outputs of PEH and TEH, power and energy densities per unit cost, and aspects to be considered in real implementation.

### 5.1. Gap Width and Electrical Output

It was found that the electrical output of either PEH or TEH was directly proportional to the gap width between the cover plate and the stopper. They tended to generate a higher electrical output (voltage, current, power, and energy) at a larger gap width. In this study, some pressing frequencies highly excited the piezoelectric cantilever, inducing an electrical output of PEH (Figure 5), but some other frequencies depressed it because of their out-of-phase nature to the resonance-frequency [44]. Even though the generated piezoelectric power (Figure 6) was much higher than the generated triboelectric power (Figure 9), PEH power output depended more strongly on the resonance frequency of its structure than TEH did [45], limiting the practical design of a PEH structure compared with that of a TEH structure. The electrical power of TEH increased linearly with gap width and excitation (or pressing the cover plate) frequency (Figure 9). The output energy of TEH was more stable than that of PEH, because TEH does not depend on the resonance frequency of its structure, as presented in Figure 10. This stability was observed in the experimental outcomes as a linear dependence of electrical output with excitation frequency.

The tested gap widths were limited to 4 mm because, when it was larger than that, the cantilever of PEH would overbend, and the tip mass would hit the cover plate and the base, leading to severe damage to the cantilever. In contrast, the TEH structure benefited from a wider gap width, within a limit. A wider gap width of no more than ten times the thickness of tribo-material would generate a higher power from this energy harvester [46]. The final velocity with which the two triboelectric materials touched each other, as well as the surface roughness and configuration of the two tribo-electric materials, were the main design parameters for TEH energy harvester [21,47].

### 5.2. Power and Energy Densities per Unit Cost

To usefully compare the performances of PEH and TEH, their power and energy densities should be investigated relative to their volume. Their practical applications absolutely depend on the economy of the initial investment capital. In this study, the power and energy densities were investigated at their respective maximum power, corresponding to an optimum resistance. The optimum resistance of these two energy harvesters was very different because of the different characteristics (e.g., operating frequency, number of generators, and type of electrical connection) of the two electricity-generating materials [48]. Figure 12 shows that the power density of PEH increased exponentially with gap width. On the other hand, the power density of TEH increased linearly with gap width; this related phenomenon was also reported by Niu et al. [49]. They investigated the electrical output from TEH, which is related to the gap width regarding the charging behaviour of the TEH capacitance model. Regarding cost, as shown in Figure 14, TEH provided a much higher power density for a very low cost compared with PEH. Moreover, it was easier to fabricate and could use a wide-range of triboelectric materials.

Another important condition to consider in this kind of study was the time period for electrical output data collection. Figure 13 shows the outcomes of the highest-peak energy density provided by PEH. As can be observed, the maximum energy densities of PEH achieved by different pressing frequencies were drastically different. In contrast, TEH’s maximum energy density was proportional to the pressing frequency. There was no resonance frequency effect. Figure 15, moreover, shows that the maximum energy density per unit cost increased linearly. Furthermore, TEH not only provided a much higher energy density per unit cost than PEH, but also was not sensitive to variation in ambient input parameters—that is, TEH was not sensitive to resonance excitation from human activities.

In addition, the output performance for piezoelectric and triboelectric energy harvesters is compared and summarized in Table 4; this table lists references, size of the harvester, voltage, power, power density, operating frequency, and optimal load. However, this table shows the related correspondence that we found regarding our research. These parameters (e.g., power density and optimal load) appeared to follow the same trend in our research, but the operating frequency in this table was extremely different due to our research being suitable for the floor-tile energy harvester.

### 5.3. Aspects to Be Considered in Realistic Implementation of the Energy Harvesters

When it comes to real applications, for example, the floor-tile energy harvester, optimal design parameters of TEH would be more easily changed to handle unexpected problems because the structural design and arrangement of parts of the test bench did not affect its resonance frequency. Our study, on the other hand, addressed a different issue. We focused on an upscale application of converting the force from human walking steps in a crowded area into electricity, that is, a floor-tile energy harvester. As listed in Table 5, the advantages and disadvantages of PEH and TEH in this scenario can function as the main design factors of a more modern version of these energy harvesters. It should be noted that the detail in Table 5 gives the guideline for the selection of transducers for a floor tile energy harvester, while other designs may rank the trade-offs differently.

## 6. Conclusions

This study developed two types of energy harvesters based on direct force excitation. Piezoelectric and triboelectric energy harvesters (PEHs and TEHs) were newly designed to be embedded in floor tiles. Test benches of a piezoelectric energy harvester (PEH) and a triboelectric energy harvester (TEH) were developed with the same kind of mechanical actuator and excitation parameters. From the outcomes of electrical performance experiments, PEH was shown to provide higher power and energy densities than those of TEH, and those densities were dependent on the induced displacement gap between the cover plate (mechanical actuator) and the stopper as well as the actuating frequency. PEH was better in this regard than TEH because the piezoelectric material mounted on the cantilever of PEH could be bent to a high degree, and hence was able to provide high power. However, even though PEH provided higher power and energy densities in absolute terms, TEH provided higher power and energy densities per unit cost. TEH may be a better alternative for the future because the costs of triboelectric materials were much lower than the costs of piezoelectric materials. Future work can be on investigating and designing multiple triboelectric material strips in a generator to boost output power or an investigation of a hybrid design [59,60] to take full advantage of the strengths of both types of electricity-generating materials.

## Figures and Tables

**Table 1:** Physical and electrical properties of the piezoelectric material.

Property (Unit)	Value
Density *ρ* (Kg/m^3^)	7800
Mechanical Q (*Q_m_*)	60
Elastic (Young’s modulus) Y^E^_3_ (N/m^2^)	5 × 10^10^
Relative dielectric constant *K^T^_3_* (@ 1 kHz)	2100
Piezoelectric coefficient *d_33_* (pC/N)	500
Piezoelectric coefficient *d_31_* (pC/N)	−210
Piezoelectric voltage constant g_33_ (Vm/N)	23 × 10^−3^
Piezoelectric voltage constant g_31_ (V-m/N)	−10.4 × 10^−3^
Coupling coefficient *K_33_*	0.74
Coupling coefficient *K_31_*	0.37
Polarizing field (*E_p_*) V/m	>1.7 × 10^6^
Initial depolarizing field *E_c_* (V/m)	~4 × 10^5^
Coercive field *E_c_* (V/m)	~1.0 × 10^6^

**Table 2:** Physical properties of the polytetrafluoroethylene (PTFE) sheet.

Property (Unit)	Value
Density (g/cm^3^)	2.3–2.45
Water absorption (%)	>0.01
Tensile strength (kg/cm^2^)	140–350
Flexural strength (kg/cm^2^)	16.4
Rockwell hardness	D55
Izod impact strength (kg cm/ cm with notch)	2.5–2.7
Friction coefficients	0.10–0.04
Coefficient of linear thermal expansion (x 10^−5^/°C)	7.0–10.0
Thermal conductivity (kcal/m. Hr. °C)	6.0
Heat distortion temperature (°C)	120
Heat resistance (°C)	–70–260
Dielectric breakdown strengths (kV/mm)	19
Coefficient of volume resistance (Ohm-cm)	10^18^

**Table 3:** Power consumption of common small electronic devices.

Device	Power Consumption	PEH	TEH
Watches [39]	3–10 µW	applicable	applicable
Smoke detector [40]	4.95 µW	applicable	applicable
Peacemakers [39]	25–80 µW	applicable	applicable
Capacitive strain gauge [41]	600 µW	applicable	applicable
Hearing aids [42]	<1.4 mW	applicable	applicable
CO_2_ sensor [43]	<3.5 mW	applicable	not applicable
Digital clocks [39]	13 mW	applicable	not applicable
Light-emitting diode [39]	25 mW–100 mW	applicable	not applicable

**Table 4:** Output performances of PEH and TEH.

Reference	Harvester Size	Voltage (V)	Power	Power Density	Frequency (Hz)	Optimal Load
1. Piezoelectric energy harvester						
Panthongsy et al. [1]	2.80 cm^3^	7.57	0.58 mW	2.07 mW/cm^3^	20.83	99 kΩ
Yang et al. [50]	241 mm^3^	22.50	2.53 mW	10.50 mW/cm^3^	44.00	200 kΩ
Dai et al. [51]	2.26 cm^3^	24.47	1.06 mW	0.47 mW/cm^3^	51.00	564.7 kΩ
Sriramdas et al. [52]	108 mm^3^	3.96	8.1 µW	0.08 mW/cm^3^	30.80	1 MΩ
Ma et al. [53]	270 mm^3^	1.46	3.18 µW	0.012 mW/cm^3^	93.00	700 kΩ
Dhakar et al. [54]	102.08 mm^3^	6.32	40 µW	0.39 mW/cm^3^	36.00	1 MΩ
Lee et al. [55]	0.023 mm^3^	0.84	1.38 µW	61.3 mW/cm^3^	255.9.	510 kΩ
Song et al. [56]	0.11 mm^3^	0.03	0.023 µW	0.209 mW/cm^3^	48.00	40 kΩ
Zou et al. [57]	400 mm^3^	12.29	387 µW	0.968 mW/cm^3^	9.90	390 kΩ
Morimoto et al. [58]	0.26 mm^3^	0.51	5.3 µW	20.46 mW/cm^3^	126.00	50 kΩ
2. Triboelectric energy harvester						
Zhang et al. [5]	18.75 cm^2^	36	162 µW	86.4 mW/cm^2^	-	8 MΩ
Jurado et al. [6]	64 cm^2^	3.83	307.88 µW	19.24 µW/cm^2^	150	10 MΩ
Shamsuddin et al. [17]	118.16 cm^2^	40.00	17 µW	0.14 µW/cm^2^	-	60 MΩ
Yang et al. [18]	36 cm^2^	749.40	9.36 mW	260 µW/cm^2^	3.2	60 MΩ
Uddin et al. [19]	2.28 cm^2^	16.20	36 µW	15.8 µW/cm^2^	3	1 MΩ
Xia et al. [26]	6 cm^2^	48.00	2.88 mW	480 µW/cm^2^	-	800 kΩ
Kim et al. [27]	36 cm^2^	13	210 µW	48 µW/cm^2^	-	20 MΩ
Mule et al. [29]	4 cm^2^	774.59	10 mW	2.54 mW/cm^2^	-	60 MΩ

**Table 5:** Advantages and disadvantages of PEH and TEH.

	Piezoelectric Energy Harvester	Triboelectric Energy Harvester
**Advantages**	- Low internal resistance - Two stages of output voltage generation - High current, power, and energy density - Low output voltage (able to power small electronic devices)	- Simple construction - Easy fabrication - Smaller displacement than that required by the piezoelectric energy harvester - Low cost - Two stages of output voltage generation - Energy density is proportional to pressing frequency
**Disadvantages**	- Complex construction - High cost of material - Low output voltage - Pressing frequency may affect energy density - Large gap displacement may over-bend the cantilever	- High internal resistance - Low current, power, and energy densities - Environmental condition may affect its electrical output characteristics - High output voltage (in term of power management circuit design)
